# Case Report: TEMPI syndrome: Report of three cases and treatment follow-up

**DOI:** 10.3389/fonc.2022.949647

**Published:** 2022-08-03

**Authors:** Zhuo-Fan Xu, Jing Ruan, Long Chang, Sijin Wu, Jinkai Lin, Wei Wang, XinXin Cao, Lu Zhang, Jian Li, Daobin Zhou, Wei Zhang

**Affiliations:** ^1^ Department of Hematology, Peking Union Medical College Hospital, Beijing, China; ^2^ School of Medicine, Tsinghua University, Beijing, China

**Keywords:** TEMPI syndrome, monoclonal gammopathy, erythrocytosis, plasma cell disorder, case report

## Abstract

The TEMPI syndrome is a novel and rare disease with five distinct clinical features: Telangiectasis, Erythrocytosis, Monoclonal gammopathy, Perinephric fluids collection, and Intrapulmonary shunting. Here, we report three cases of TEMPI syndrome and their treatment response. The three patients were presented to our department with polycythemia, abdominal distension, and dyspnea. On admission, all patients manifested telangiectasis, erythrocytosis, monoclonal gammopathy, and intrapulmonary shunting. Patient 1 and 2 manifested perinephric fluids collection. In addition, all patients had skin pigmentation, patient 1 and 2 had polyserosal effusion, two symptoms that had not been associated with TEMPI syndrome before. The three patients showed various response to plasma cell-directed therapy. We demonstrated their treatment response by measuring erythropoietin, hemoglobin, and M protein levels throughout therapy. This report suggested that TEMPI syndrome is a rare yet treatable disease. The diagnosis and treatment of it remain challenging.

## Introduction

The TEMPI syndrome is a rare disorder characterized by Telangiectasis, Erythrocytosis, Monoclonal gammopathy, Perinephric fluids collection, and Intrapulmonary shunting. First described by Sykes et al. in 2011 (1), there are only 29 cases reported worldwide (1–11). Herein, we report three new cases of TEMPI syndrome diagnosed in our department. We presented comprehensive clinical evaluations and relevant history of the patients and highlighted the possible association between skin pigmentation and TEMPI syndrome. Two of the patients showed favorable long-term treatment response. We demonstrated their response by monitoring the biochemical markers throughout therapies.

## Case presentation

### Patient 1

A 60 years old male with a five–year history of polycythemia was presented to our department in 2020. For the past five years, his polycythemia had been presumed to be due to polycythemia vera (PV). He was treated with hydroxyurea and phlebotomy, to which he reported moderate response and frequent recurrence. In 2019, he tested positive for IgA-κ and was suspected of multiple myeloma. The patient came to our department for further evaluations.

On admission, he had an oxygen saturation (SpO2) of 94%. Physical exams showed telangiectasis on his face, chest, and back. Skin pigmentation was found on his face and lips. His blood tests demonstrated polycythemia (RBC 7.13×10^12^/L, HGB 151g/L). Laboratory findings included increased EPO level (676.16 mIU/mL) and monoclonal gammopathy (M protein 5.10g/L, IgA-κ(+)). Additionally, increased IL-6, IL-8, and TNFα levels were detected. A bone marrow biopsy showed 1.5% of plasma cells. Enhanced computed tomography (CT) revealed moderate amount of perinephric fluid and small amount of abdominal and pelvic fluid. Doppler bubble test indicated intrapulmonary shunt, while the result of lung perfusion scintigraphy (LPS) with Tc-99m MAA was unremarkable. (**Table 1**)

**Table 1:** Characteristics of patients with TEMPI syndrome.

Characteristic	Patient 1	Patient 2	Patient 3
Demographic			
Age (yr)	60	57	57
Gender	Male	Male	Female
First presentation	Polycythemia	Abdominal bloating	Dyspnea
TEMPI syndrome			
Telangiectasias	Face, chest, back	Chest, back	Neck, chest
Erythrocytosis and EPO			
RBC (×10^12^/L)	7.13	7.32	4.73
HGB (g/L)	151	175	181
HCT (%)	52.3	61.6	55.9
EPO (mIU/mL)	676.16	>741.00	209.77
Monoclonal gammopathy			
M protein (%)	6.90%	12.00%	35.40%
M protein (g/L)	5.1	9.6	NR
Type	IgA-κ	IgG-κ	IgG-λ
Light chain	sFLC-λ↑	sFLC-κ↑	sFLC-λ↑
κ/λ	0.528	2.059	0.483
Perinephric fluid	+	+	–
Intrapulmonary shunting			
SpO2	94%	93%	89%
Bubble test	+	+	+
LPS	–	Lung telangiectasis	–
Shunting fraction	–	21.10%	10.40%
Others			
Venous thrombosis	–	–	–
Serous effusion	Abdominal, pelvic	Chest, abdominal, pelvic	–
Inflammation markers	IL-6↑, IL-8↑,TNFα↑	–	TNFα↑
Bone marrow plasma cells	1.50%	2.50%	10.50%
Skin pigmentation	+	+	+

**Figure 1:**
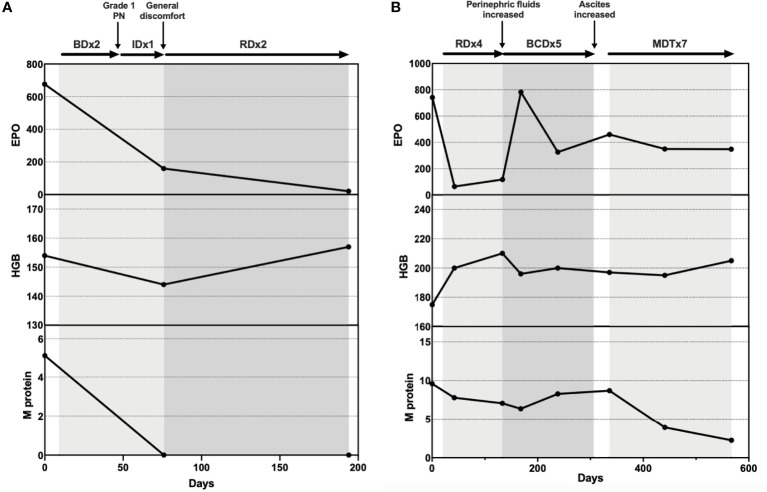
**(A)** Treatment response of Patient 1; **(B)** Treatment response of Patient 2.

Meeting all major criteria (telangiectasis, elevated EPO and erythrocytosis, monoclonal gammopathy) and minor criteria (perinephric fluid, intrapulmonary shunting) (12), the diagnosis of TEMPI syndrome was established. The patient was initiated on plasma cell-directed therapy with bortezomib and dexamethasone (Bd). After two courses of Bd, he developed peripheral neuropathy (grade I), with no obvious decrease in EPO level. Ixazomib and dexamethasone (Id) were then prescribed. After one course of Id, there was a significant decrease in EPO and HGB levels. However, the patient reported itchiness and general discomfort. Ixazomib was discontinued, and therapy of lenalidomide and dexamethasone (Rd) was initiated. After two courses of Rd, he demonstrated satisfactory response: The telangiectasis on his face and chest improved substantially; EPO fell within the normal range; M protein was tested negative; Perinephric fluids were decreased. (**Figure 1**)

### Patient 2

A 57 years old male with a seven-year history of skin pigmentation and a three-year history of abdominal distension was presented to our department in 2020. The patient developed facial pigmentation and lips cyanosis in 2013. He was hospitalized for diarrhea, abdominal distension, and fatigue in 2017. His laboratory and endoscopy examinations were unremarkable except for elevation of RBC and HGB levels. The patient was treated with supportive medicine for diarrhea and hydroxyurea for suspicious PV. During treatment, his diarrhea improved, his HGB level decreased, while his abdominal distension and fatigue remained. In 2018, the patient started to suffer recurrent upper respiratory infection and dyspnea. His CT images suggested chest and abdominal fluids collection. In 2020, he experienced progressive abdominal distension and came to our department for further examination.

**Figure 2:**
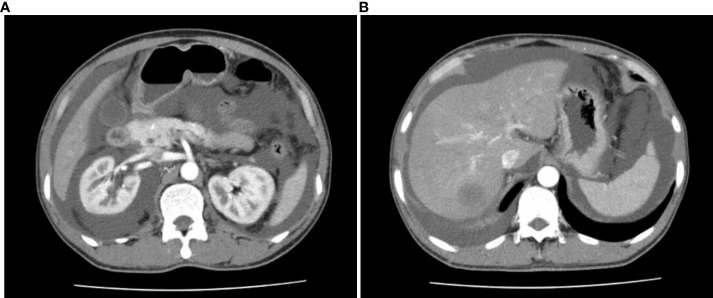
Enhanced CT scans of patient 2. **(A)** Perinephric fluids; **(B)** Abdominal fluids.

On admission, he had a SpO2 of 93%. His physical exams showed whole-body skin pigmentation, white nails, and telangiectasis on the chest, back, and oral mucosa. He was positive for abdominal distension signs and shifting dullness. Lab tests revealed elevated HGB (175g/L) and EPO levels (>741.00mIU/mL), and monoclonal gammopathy (M protein 9.60g/L, IgG-κ(+)). Bone marrow biopsy showed 2.5% plasma cells. Enhanced CT revealed extensive perinephric, abdominal, and pelvic fluids. (**Figure 2**) Ascites puncture aspirated 800mL transudate ascites with normal cell counts and protein level. Arterial blood gas (ABG) test indicated right-to-left shunting (pO2 60mmHg, pO2(A-a)e 54.3mmHg, FShumte 31.9%). Doppler bubble test showed intrapulmonary shunting. LPS demonstrated pulmonary telangiectasis. First-pass LPS confirmed intrapulmonary shunting (shunt fraction 21.1%). (**Table 1**)

The diagnosis of TEMPI syndrome was established for all major and minor criteria were fulfilled. After four courses of Rd, the patient demonstrated mixed response. While his dyspnea and abdominal distension improved and EPO level dropped, his HGB and M protein levels remained high, and his perinephric fluids collection increased. He was then given bortezomib, cyclophosphamide, and dexamethasone (BCD) for further treatment. After three courses of BCD, the patient experienced dizziness, dyspnea, and aggravated abdominal distension. He went through ascites drainage and discontinued BCD therapy for 1 month. Lab tests showed increased EPO, HGB, and M protein levels. The patient then started melphalan, dexamethasone, and thalidomide (MDT) therapy. He responded well to MDT. After seven courses, the telangiectasis on his chest became less prominent, and his EPO and M protein levels dropped continuously. However, his RBC counts and HGB level remained high. (**Figure 1**)

### Patient 3

A 57 years old female with a seven-year history of dyspnea was presented to our department in 2021. The patient had been experiencing dyspnea and dizziness after physical activities since 2014. Four years later, in 2018, she developed rashes on her neck and chest. In 2020, she was hospitalized for dyspnea. Her pulmonary function test indicated type I respiratory failure and asthma. Her blood tests showed polycythemia and monoclonal gammopathy. Her bone marrow biopsy was normal. She was then initiated on asthma treatment and hydroxyurea treatment, to which she showed no response. During treatment, her dyspnea worsened. She was referred to our department for evaluation.

**Figure 3:**
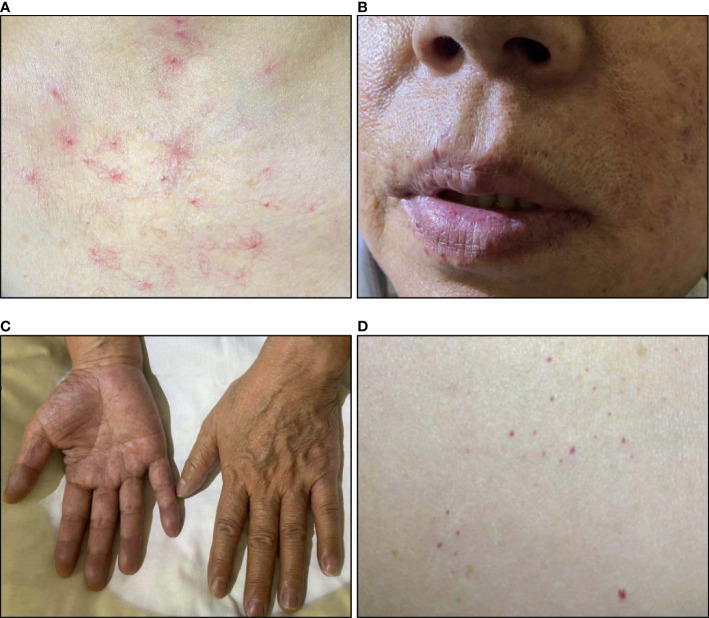
Skin changes of patient 3. **(A)** Telangiectasis on the neck and chest; **(B)** Telangiectasis on lips; **(C)** Cyanosis of fingers; **(D)** Rash on the abdomen.

On admission, she had a SpO2 of 89%. Physical exams showed telangiectasis on her neck, chest, and abdomen. Peripheral cyanosis was found on the extremities. Skin pigmentation was found on her face and extremities. (**Figure 3**) Elevation of HGB level (181g/L) and monoclonal gammopathy (M protein% 35.4%, IgG-λ(+)) were confirmed by laboratory tests. Her EPO level was 209.77mIU/mL. Her TNFα level was elevated as well. Bone marrow biopsy revealed 10.5% plasma cells. Abdominal CT showed no sign of perinephric fluids. Chest CT indicated emphysema and possible interstitial lung disease. First-pass pulmonary perfusion scintigraphy confirmed intrapulmonary shunting (shunt fraction 10.4%).

The patient was diagnosed with TEMPI yndromee for three major and one minor criteria were met. Bd therapy was initiated. Her EPO (209.77 –> 56.55mIU/mL) and M protein (35.4% -> 7.2%) levels dropped dramatically after one course of Bd. Her HGB levels (181 –> 140g/L) and RBC counts (4.73 –> 4.32x10^12^/L) also fell within the normal ranges. However, she developed swelling and pain in the right calf, which later progressed into cellulitis. Ultra-sound showed no sign of venous thrombosis. After abscess incision and drainage, the patient is now under skin grafting and careful monitoring of TEMPI syndrome.

## Discussion

The TEMPI syndrome is a rare multisystem disease with unique cutaneous features and a signature set of clinical manifestations. It was first described in a case series of 6 patients in 2011 (1), where they shared five characteristics — telangiectasias, elevated EPO level and erythrocytosis, monoclonal gammopathy, perinephric fluid collections, and intrapulmonary shunting. Later, more cases were identified and reported as individual case reports across the globe (12), and TEMPI syndrome was officially categorized as a plasma cell disorder with paraneoplastic manifestations. Zhang et al. reviewed the first 15 published cases in 2018. It appears that TEMPI syndrome is an acquired disorder that slowly progresses and generally manifests after middle age. No gender, ethnicity, or geography predisposition was reported (13).

Almost all patients manifested telangiectasias, erythrocytosis, and monoclonal gammopathy, the three major criteria of TEMPI syndrome (12). The three patients we reported met all major criteria of TEMPI syndrome, although with distinct onset symptoms (Asymptomatic polycythemia; Skin pigmentation and abdominal distension; Dyspnea). Even though the diagnostic features are unique, misdiagnosis of PV, renal, pulmonary, or hematologic disorders appeared almost invariably throughout the cases. All of our patients had been misdiagnosed with PV and received hydroxyurea or phlebotomy treatment. The time between their disease onset and diagnosis of TEMPI syndrome spans from 5 to 7 years. Despite the rareness of the disease, early recognition and diagnosis are important. Proper treatment could prevent the progressive course of EPO increment, and the progression to more severe symptoms such as intrapulmonary shunting and perinephric fluids. Improper treatment with hydroxyurea and phlebotomy could lead to iron deficiency and microcytosis (12). From 2020 to 2022, our department has already diagnosed 3 new cases of TEMPI syndrome, which make us suspect there is more TEMPI patient unidentified. Early recognition and proper diagnosis of this disease require more awareness from hematologists, dermatologists, and general practitioners.

The less predominant clinical features and associated findings of TEMPI syndrome include venous thrombosis, spontaneous intracranial hemorrhage, liver hemangiomas, diarrhea, ascites, and pleural effusion (13). These sporadic symptoms are mostly considered coincidental. However, as the number of cases continues to grow, more associated symptoms might be discovered.

In our cases, patient 2 was presented with polyserosal effusion and a history of diarrhea, abdominal distension, and dyspnea. After careful examinations, no underlying diseases were found responsible for his polyserosal effusion. Patient 1, to a lesser extent, also had asymptomatic abdominal and pelvic fluids. It aroused our curiosity whether chest and abdominal fluids were associated with TEMPI syndrome. During the treatment of patient 2, a remarkable drop in M protein was observed, however, we did not see a simultaneous resolution of either chest and abdominal fluids or perinephric fluids.

We also noticed that all three patients demonstrated diffusive or localized skin pigmentation. Hyperpigmentation is commonly seen in patients with POEMS syndrome, yet has not been reported in TEMPI syndrome. Patient 1 was found to have localized facial pigmentation on admission. Patient 2 reported a seven–year history of whole-body skin pigmentation starting from the face and lips. Patient 3 was found to have diffusive skin pigmentation on admission. We wonder whether hyperpigmentation was one of the skin changes possibly associated with TEMPI syndrome that had been overlooked in the past. In POEMS syndrome, VEGF is a proposed candidate for contributing to skin changes (14). Increased VEGF and IL-6 levels were described in the previous cases (4). We did not measure VEGF level, but we did notice elevation of IL-6, IL-8, and TNFα in patient 1 and elevation of TNFα in patient 3. For patient 1 and 2, there were substantial improvements in telangiectasis during treatment. However, there was no self-reported improvement in skin pigmentation. For patient 3, no obvious change of either telangiectasias or skin pigmentation was observed during the two–month follow–up period. Pictures of her skin changes were documented for future reference.

Besides the clinical symptoms, little is known about the etiology and pathogenesis of TEMPI syndrome. One theory postulates that the syndrome is an autoimmune disease caused by antibodies, chemokines, or cytokines secreted by monoclonal plasma cells since plasma cell–directed therapy could reverse the manifestations (12). Khan et al. proposed that HIF-1α may play a role in the pathogenesis of TEMPI syndrome as well. Since bortezomib also affects the function of HIF-1α, inhibition of HIF-1α could also explain the resolution of symptoms (15). More recently, Sun et al. found that the expression of macrophage migration inhibitory factor (MIF) was significantly upregulated in three patients with TEMPI syndrome (16). Altogether, these hypotheses and findings suggest that multiple pathways might be involved in the pathophysiology of TEMPI syndrome. Due to the rareness of the disease, uncovering its etiology and pathogenesis will require international collaboration in collecting samples and data. We have not been able to collect research samples of the three patients, but we are open to sharing clinical data and possible research samples in the future.

Up till now, plasma cell-directed treatment is the only first–line therapeutic option for TEMPI syndrome (12). The complete and partial responses gained by bortezomib-based regimens have been revealed in multiple case reports (17–19). The alternative therapies that have been reported include daratumumab (20), lenalidomide (9), and autologous transplantation (21). Rapid and remarkable treatment response was seen in several patients. All symptoms of TEMPI syndrome appeared to be reversible under proper therapy (18). However, recurrence and refractory were also reported in multiple cases (7–9). Due to the lack of therapeutic experience, there are currently no treatment guidelines for those patients. Our limited experience suggested that personalized regimens need to be chosen and adjusted for patients by carefully monitoring their response. In our cases, Patient 1 reached satisfactory response with lenalidomide, while Patient 2’s perinephric fluids progressed after four courses of Rd. Patient 2 was then treated with BCD, followed by MDT. After seven courses of MDT, a substantial drop in M protein and resolution of telangiectasia and serous fluids were finally observed. Patient 3 demonstrated rapid and dramatic response to bortezomib, but she developed infection and cellulitis after one course of Bd.

There is currently no prognostic marker for TEMPI syndrome. Since monoclonal gammopathy has been proposed to be the cause of TEMPI syndrome, M protein levels are carefully monitored throughout therapy. However, no clues of correlation between initial M protein level and treatment response was found. In patients who relapsed after initial therapy, the increase of EPO level seems to be the most sensitive marker (12). Nevertheless, EPO level before treatment did not predict disease severity or treatment response either. Up till now, the follow–up times are still too short to conclude long-term survival. Understanding the prognosis and outcomes of this rare disease will depend on the continued description of patients worldwide.

In conclusion, TEMPI syndrome is a rare disorder that can be reversible with timely diagnosis and appropriate treatment. We herein report the clinical presentation and treatment response of three new cases of TEMPI syndrome. Our observation showed that the possible associated symptoms and therapy responses varied among patients. More studies are in need to explore the etiology, pathogenesis, clinical manifestations, treatment, and prognosis of this ultra-rare disease.

## Data availability statement

The original contributions presented in the study are included in the article/supplementary material. Further inquiries can be directed to the corresponding authors.

## Ethics statement

Written informed consent was obtained from the participant for the publication of this case report.

## Author contributions

Z-FX drafted the manuscript. All authors participated in patient management and data collection. JR, LC, and SW contributed to the preparation of figures. All authors contributed to the article and approved the submitted version.

## Acknowledgments

Thanks are given to all the clinicians providing care and management to the patients.

## Conflict of interest

The authors declare that the research was conducted in the absence of any commercial or financial relationships that could be construed as a potential conflict of interest.

## Publisher’s note

All claims expressed in this article are solely those of the authors and do not necessarily represent those of their affiliated organizations, or those of the publisher, the editors and the reviewers. Any product that may be evaluated in this article, or claim that may be made by its manufacturer, is not guaranteed or endorsed by the publisher.
